# Use of the Alexis® Wound Protector for Exposure and Sterility in Perianal Oncologic Surgery: A Technical Report

**DOI:** 10.7759/cureus.104190

**Published:** 2026-02-24

**Authors:** Samer Doughan, Mohamad El Haress, Ahmad Jradi

**Affiliations:** 1 Department of Surgery, American University of Beirut Medical Center, Beirut, LBN; 2 Faculty of Medicine and Medical Sciences, University of Balamand - Koura Campus, Balamand, LBN

**Keywords:** alexis wound retractor, gastrointestinal stromal tumor, perianal tumor, sphincter preservation, surgical exposure, surgical technique

## Abstract

Surgical excision of perianal tumors presents significant technical challenges due to limited exposure, proximity to the anal sphincter complex, and an increased risk of contamination from the anal canal. Adequate visualization while preserving sphincter function and maintaining sterility is essential to ensure safe oncologic resection. Conventional retraction methods may provide suboptimal exposure in this anatomically constrained region. We describe a technical adaptation using the Alexis® wound retractor (Applied Medical Resources Corporation, Rancho Santa Margarita, California, United States) to optimize exposure and maintain sterility during perianal oncologic surgery in a patient with a right perianal extragastrointestinal stromal tumor (EGIST) involving the external anal sphincter. After initial dissection and identification of sphincter anatomy, a small Alexis wound retractor was inserted into the perianal incision, providing circumferential 360-degree exposure while acting as a physical barrier between the surgical field and the anal canal. The adjustable depth and flexible design of the device allow controlled retraction during deep dissection in the intersphincteric plane, facilitating precise tumor mobilization and en bloc resection while protecting the external sphincter. This technique improves visualization, reduces reliance on hand-held retractors, and supports sphincter-preserving dissection in appropriately selected cases. In the present case, no surgical site infection occurred, sphincter continence was preserved, and the patient was discharged on postoperative day 3. The use of the Alexis wound retractor represents a simple and reproducible technical modification that addresses both exposure and sterility challenges in perianal oncologic surgery and may facilitate safe tumor resection with preservation of sphincter integrity. These findings are preliminary and based on a single case; prospective evaluation is needed to validate broader applicability.

## Introduction

Perianal tumors encompass a heterogeneous group of neoplasms arising from the soft tissues surrounding the anal canal, including gastrointestinal stromal tumors (GISTs), squamous cell carcinoma, adenocarcinoma of anal gland origin, and rare mesenchymal tumors [1]. Anatomically, these lesions are bounded superiorly by the levator ani, laterally by the ischiorectal fat, and medially by the internal and external anal sphincters. Tumors arising in this region often extend into or abut the intersphincteric plane, the anatomical corridor between the internal and external sphincters, making oncologically sound resection technically demanding [2]. Achieving negative surgical margins while avoiding inadvertent sphincterotomy or injury to the pudendal neurovascular bundle requires meticulous dissection in a narrow, curved operative field with limited direct visualization.

Surgical resection of perianal malignancies presents unique technical challenges that distinguish these procedures from other pelvic operations. The anatomical constraints of the perianal region, combined with proximity to the anal sphincter complex and rectum, create significant difficulties in achieving adequate surgical exposure while maintaining oncologic principles. Poor visualization of the operative field frequently compromises the surgeon's ability to perform complete tumor resection with safe margins, potentially affecting long-term oncologic outcomes [1,2].

Traditional approaches to perianal tumor excision typically employ hand-held retractors or self-retaining retractor systems. However, these conventional methods have inherent limitations. Hand-held retractors require an additional assistant and provide inconsistent exposure, while rigid self-retaining systems may not conform adequately to the curved perianal anatomy. Furthermore, maintaining sterility in the perianal region presents an additional challenge, as the proximity to the anal canal increases the risk of contamination of the surgical field [3,4].

Several retraction strategies have been described for perianal and perineal surgery. The Lone Star® retractor (CooperSurgical Inc., Trumbull, Connecticut, United States) is widely used in anorectal procedures and provides radial tension through elastic stays, but its open design exposes the anal canal, offering limited protection against fecal contamination [3]. Its radial hook-based design also provides no depth-adjustable circumferential retraction, limiting its utility for deep intersphincteric dissection. Of additional oncologic concern, perineal skin recurrence at Lone Star hook sites has been reported, raising questions about its suitability in malignant cases [5]. Improvised alternatives, such as radially placed skin sutures, lack circumferential uniformity and may obscure the operative field [6]. Regarding sterility, existing strategies have relied primarily on preoperative mechanical bowel preparation, antiseptic irrigation, and draping, without a dedicated physical barrier between the anal canal and the resection site [4]. None of these approaches simultaneously addresses the dual challenges of circumferential deep-plane exposure and active contamination prevention, particularly when dissection enters the intersphincteric plane in close proximity to the anal mucosa.

The Alexis® wound retractor (Applied Medical Resources Corporation, Rancho Santa Margarita, California, United States) was originally designed for laparotomy and laparoscopic-assisted procedures to provide circumferential wound protection and retraction. Its flexible ring design and adjustable depth have made it valuable in various surgical applications. However, its utility in perianal oncologic surgery has not been extensively described in the literature.

Ring-type wound retractors have been investigated in several surgical fields where contamination and exposure are concurrent concerns. In colorectal surgery, the Alexis O-Ring wound retractor has been shown to significantly reduce surgical site infection (SSI) rates compared to conventional wound protection [7]. In urologic oncology, the Alexis device specifically was shown to reduce SSI after open radical cystectomy, a contaminated-field operation with parallels to perianal surgery [8]. Wound retractors have also been described in resection of perineal lesions; however, their use in the intersphincteric plane for oncologic purposes, where both depth-adjustable circumferential retraction and a sterile barrier are simultaneously required, has not been systematically reported. The present report is therefore distinct in three respects: (i) it applies the Alexis device specifically to the intersphincteric plane of perianal oncologic dissection; (ii) it combines the retractor with an intraluminal betadine-soaked sponge to create a dual-barrier sterility strategy; and (iii) it demonstrates the feasibility of this technique for sphincter-preserving en bloc resection in a single case of perianal extragastrointestinal stromal tumor (EGIST). We present this technical adaptation not as a wholly novel concept, but as a practical and reproducible solution to a well-recognized operative challenge that merits wider awareness.

We describe a straightforward technique utilizing the Alexis wound retractor to optimize surgical exposure and maintain sterility during perianal tumor excision. This approach addresses the specific challenges of perianal anatomy while facilitating sphincter-preserving oncologic resection.

## Technical report

The Alexis wound retractor was used in a 60-year-old female patient, who was placed in the lithotomy position under general anesthesia.

Preoperative MRI and PET-CT confirmed a right perianal EGIST arising within the right external anal sphincter, measuring 8 × 5.8 × 5.7 cm at diagnosis, without transmural invasion of the internal sphincter or ischioanal fossa. Following 10 months of neoadjuvant imatinib therapy (400 mg daily), serial imaging demonstrated marked tumor downstaging with size reduction to 4.5 cm and a 90% decline in metabolic activity (maximum standardized uptake value (SUV_max_) 25.8-3.1). Sphincter-preserving resection was deemed oncologically feasible given the excellent treatment response and the patient's strong preference for rectal preservation.

To preserve sterility, a rectal sponge soaked in betadine was inserted into the anal canal. A 5-cm curved vertical incision was made over the right perianal region overlying the tumor, and electrocautery was used to deepen the incision through the subcutaneous tissue. Digital palpation and careful dissection were used to identify the external sphincter and define the tumor margins.

**Figure 1:**
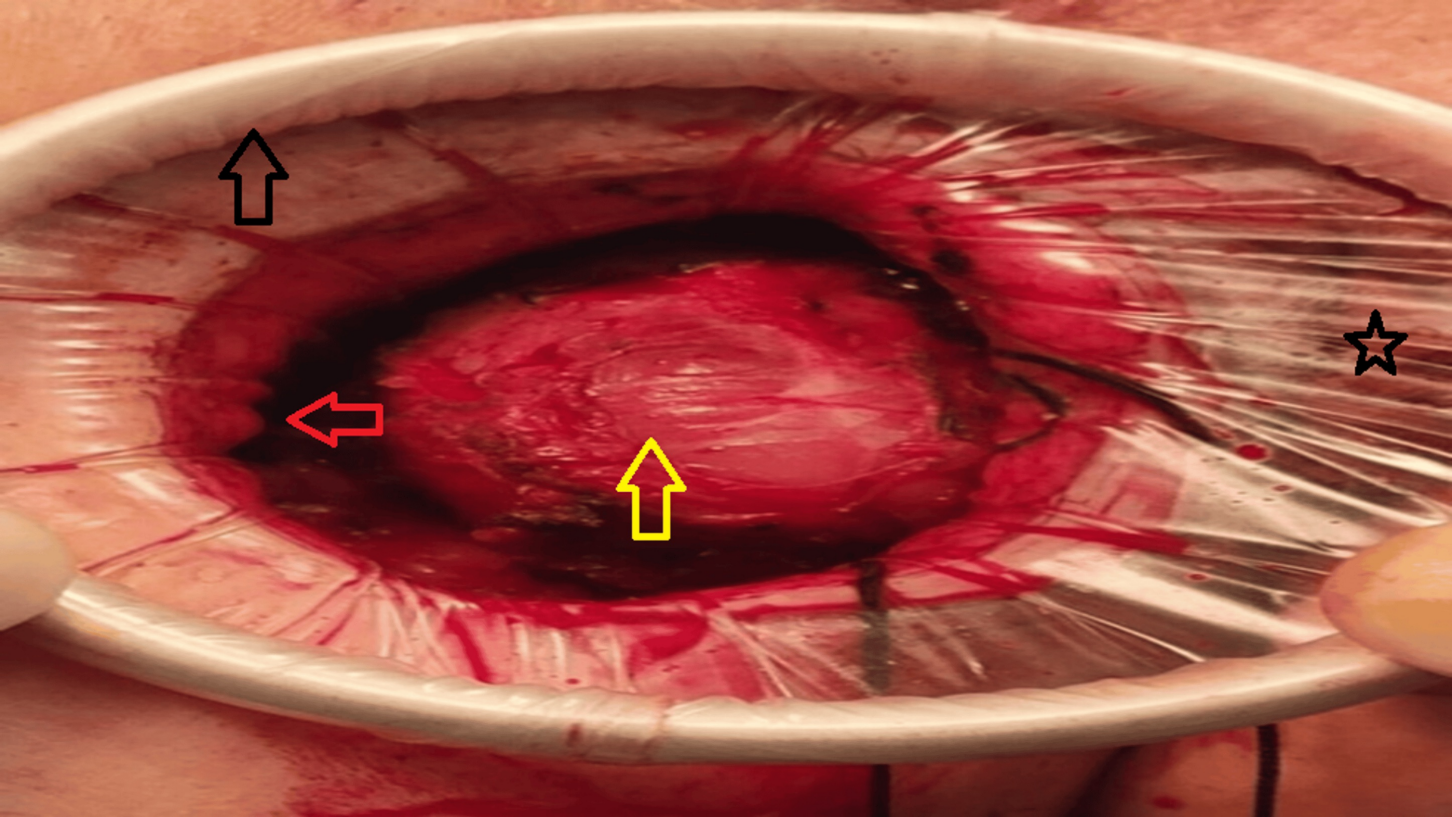
Intraoperative view showing the Alexis® wound retractor providing circumferential 360° exposure of the perianal tumor. Black arrow: outer ring of the Alexis retractor at skin level. Red arrow: inner ring of the Alexis retractor seated at depth within the wound. Yellow arrow: perianal extragastrointestinal stromal tumor with intact capsule. Star: transparent plastic sleeve of the Alexis retractor forming a sterile barrier between the operative field and the anal canal.

After limited initial dissection sufficient to identify the sphincter anatomy and establish a safe tissue plane, a small Alexis wound retractor (working diameter 2.5-6 cm), selected based on the 5-cm incision length and estimated tumor depth, was inserted into the operative field (Figure 1). 

**Figure 2:**
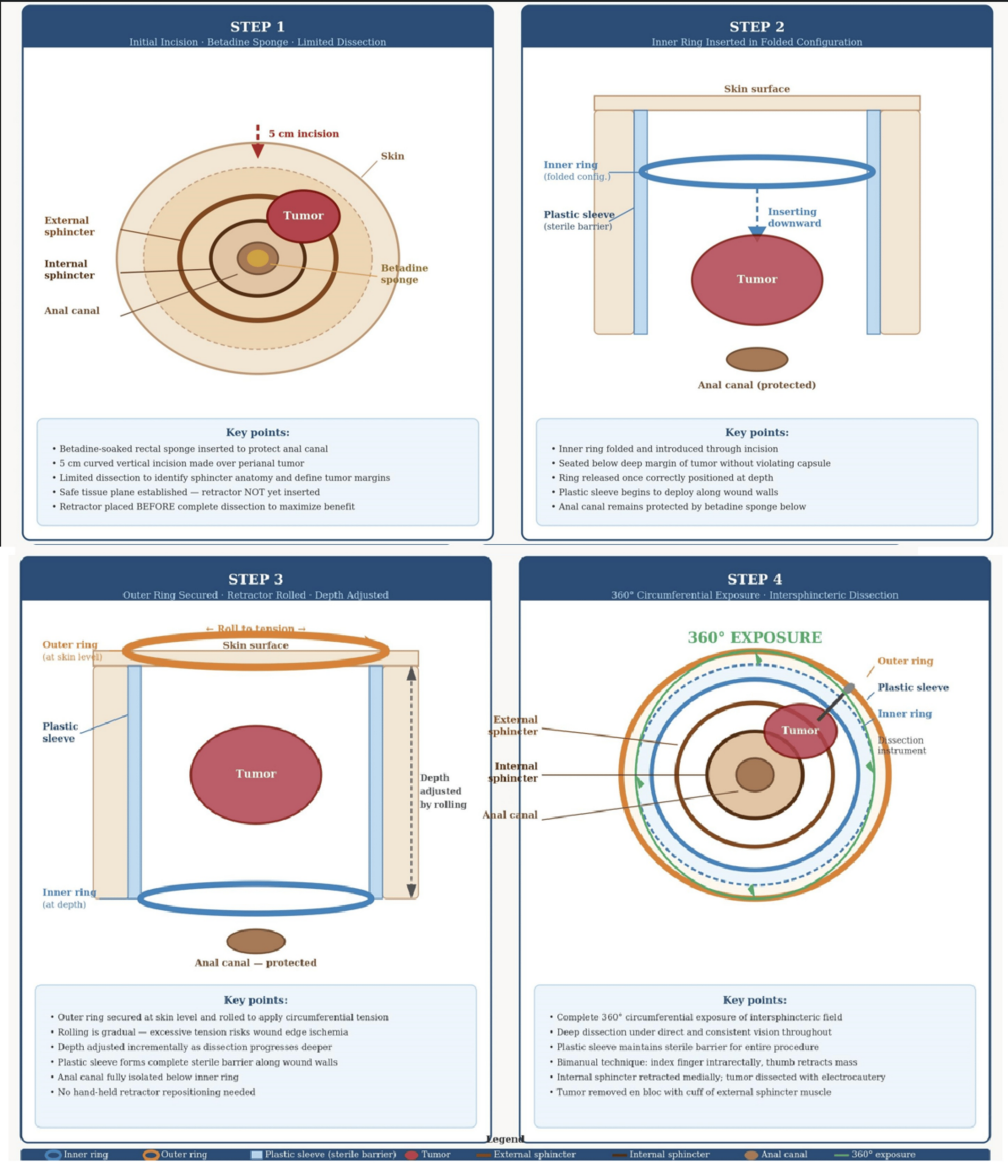
Stepwise schematic of Alexis® wound retractor placement and rolling technique. Step 1: Limited initial dissection to identify sphincter anatomy and establish a safe tissue plane, with betadine-soaked sponge in the anal canal. Step 2: Inner ring introduced in folded configuration and seated below the deep tumor margin without violating the capsule. Step 3: Outer ring secured at skin level and rolled to apply circumferential tension; plastic sleeve forms a sterile barrier to the anal canal. Step 4: Circumferential 360° operative exposure enabling deep intersphincteric dissection with continuous sterile barrier maintained throughout. Image Credit: Authors; created using Adobe Illustrator (Adobe Inc., San Jose, California, United States).

It is important to note that retractor placement preceded complete circumferential tumor dissection; the device was introduced after limited initial dissection only, so that the circumferential exposure and sterile barrier it provides could be utilized throughout the deep intersphincteric dissection rather than after its completion. The inner ring was introduced in a folded configuration, seated below the deep margin of the tumor without violating the capsule, and released. The outer ring was then secured to the skin edges, and the retractor rolled to adjust depth and tension incrementally as dissection progressed, providing optimal and consistent exposure throughout the procedure. A stepwise schematic of device placement and the rolling technique is provided in Figure 2. 

The Alexis port's circumferential 360-degree exposure allowed for deep-plane dissection while serving as a physical barrier between the surgical field and the anal canal, thereby maintaining sterility. Dissection was carried out in the intersphincteric plane, remaining close to the tumor capsule to protect the external sphincter. Using a combination of blunt and sharp dissection with right-angle instruments, the tumor was mobilized circumferentially. A bimanual technique was employed, with the index finger placed intrarectally to palpate the tumor's relationship to the rectal mucosa while the thumb retracted the mass laterally away from the internal sphincter. After the internal sphincter was retracted medially, the tumor was carefully separated from the surrounding musculature using electrocautery. Meticulous care was taken to prevent capsular breach throughout the dissection.

**Figure 3:**
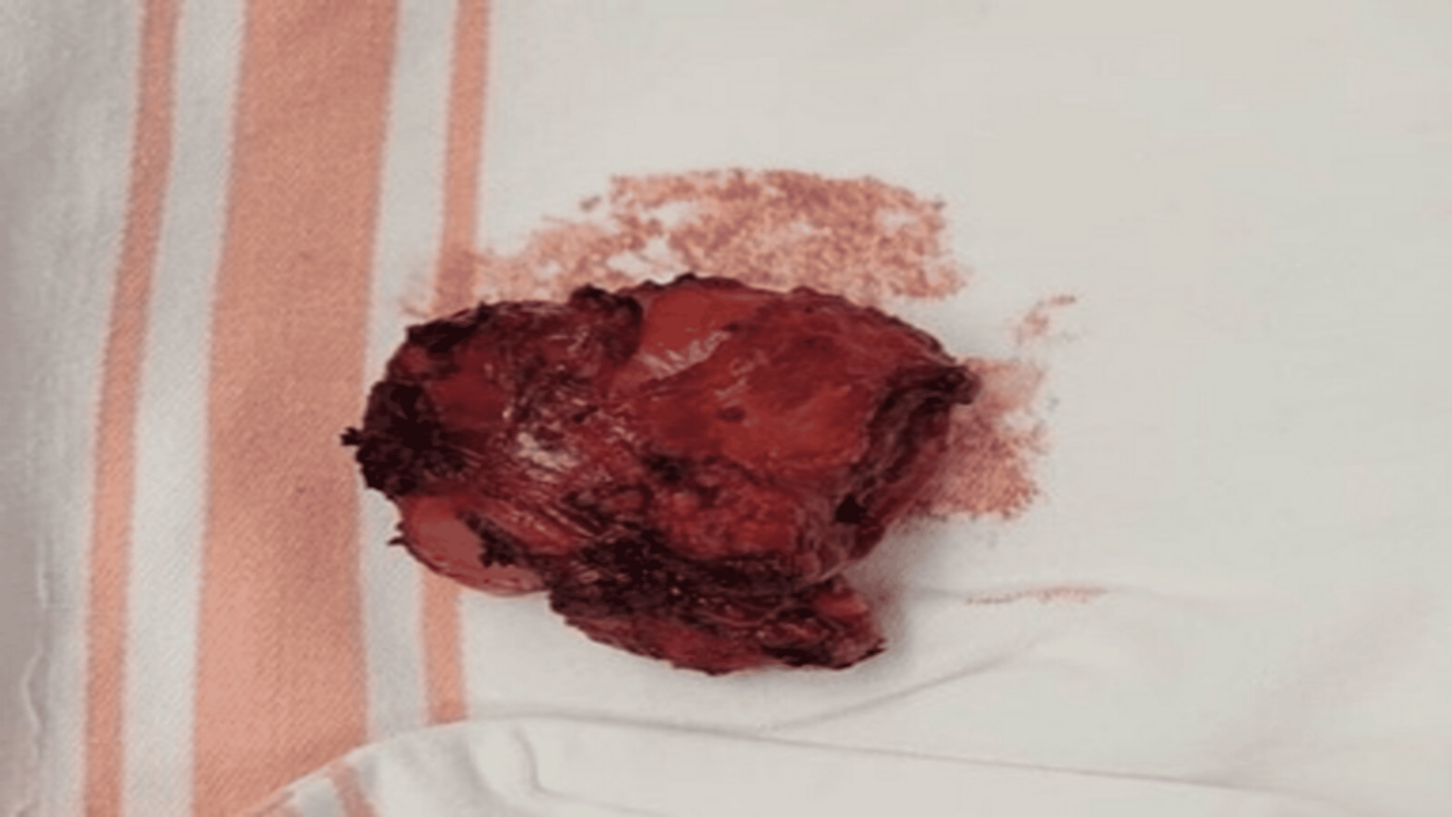
Gross specimen of the completely resected perianal tumor removed en bloc with intact capsule.

The tumor was removed en bloc with a cuff of surrounding external sphincter muscle and adjacent mucosa (Figure 3).

The external sphincter demonstrated good contractility and remained largely intact, with only a small portion of muscle resected with the specimen. The resection bed showed no obvious residual gross disease. Final histopathological examination of the 5.5-cm specimen confirmed GIST with marked treatment-related changes, 80% therapy-related fibrosis and necrosis with only 20% viable tumor remaining and minimal mitotic activity (one mitosis per 5 mm²). Margin assessment identified a microscopic tumor at inked margins in multiple foci, representing an R1 resection; precise anatomic localization of involved margins could not be determined due to the unoriented nature of the specimen.

Total operative time was 138 minutes. Estimated blood loss was 200 mL. The postoperative course was complicated by a perineal hematoma on postoperative day 2 with a hemoglobin nadir of 7.9 g/dL, managed conservatively with drain removal and compressive dressing without transfusion requirement. No surgical site infection occurred. The patient maintained continence for solid and liquid stool throughout hospitalization with no episodes of incontinence, consistent with preservation of sphincter function. She was discharged on postoperative day 3 with adjuvant imatinib 400 mg daily restarted. 

## Discussion

This technique demonstrates how the Alexis wound retractor can be effectively adapted for perianal oncologic surgery, addressing two critical challenges: surgical exposure and maintenance of sterility. The flexible design of the retractor conforms to the curved perianal anatomy, providing consistent circumferential exposure that is difficult to achieve with conventional retraction methods.

The primary advantage of this approach lies in improved visualization of the operative field. The 360-degree retraction eliminates the need for frequent repositioning of hand-held retractors and frees an assistant to participate more actively in the dissection. The adjustable depth of the retractor allows the surgeon to modify exposure dynamically as dissection progresses deeper into the pelvis. This is particularly valuable when operating in the intersphincteric plane, where precise identification of tissue planes is essential for sphincter preservation.

Several alternatives exist for perianal exposure. The Lone Star retractor, while commonly used in anorectal surgery, provides only radial two-dimensional retraction and leaves the anal canal open, offering no barrier against contamination [3,5]. In oncologic settings specifically, perineal skin recurrence at Lone Star hook sites has been reported, raising additional concerns about its suitability in malignant cases [5]. Suture-based retraction methods, while simple and cost-effective, lack the circumferential uniformity and depth control required for deep intersphincteric dissection [6]. Non-disposable ring retractors present sterilization complexity and are not designed for the curved perianal anatomy. The Alexis was selected in this setting because its flexible inner ring conforms to the perianal contour, its rolling mechanism allows dynamic depth control during progressive dissection, and its plastic sleeve provides a continuous physical barrier between the anal canal and the operative field, a combination not offered by any single alternative device [7].

Maintenance of sterility represents another significant benefit of this technique. The Alexis port creates a physical barrier between the anal canal and the surgical field, reducing contamination risk [8]. When combined with an intraluminal betadine-soaked sponge, this dual-barrier approach provides robust protection against fecal contamination, which is especially important in perianal tumor surgery, where wound complications can delay adjuvant therapy and negatively impact oncologic outcomes [9]. It should be acknowledged, however, that the evidence supporting SSI reduction with wound retractors in this specific setting is indirect. Published data on Alexis and similar devices demonstrating reduced surgical site infection rates derive primarily from colorectal resections and open radical cystectomy [7,8], operative fields that share the contamination challenge but differ anatomically from the perianal region. The present report, therefore, focuses on technical feasibility and the logical extension of these principles to perianal oncologic surgery rather than providing direct comparative SSI data.

The improved exposure facilitates more precise dissection, potentially reducing the risk of inadvertent sphincter injury. In the present case, the external sphincter demonstrated good contractility following resection, with continence preserved for both solid and liquid stool at discharge. Complete oncologic resection with clear margins remains the ideal goal in perianal malignancies, as positive margins often necessitate additional surgery or more aggressive adjuvant therapy [10]. In this case, margin assessment identified a microscopic tumor at inked margins (R1 resection), attributable in part to the unoriented nature of the specimen and the anatomical constraints of the intersphincteric plane. The profound pathologic response to neoadjuvant imatinib, with 80% therapy-related necrosis and minimal residual mitotic activity, provides oncologic reassurance and supports the continuation of adjuvant imatinib therapy as the primary strategy for disease control in this setting.

Cost-effectiveness is an additional consideration. The Alexis retractor is a single-use device that adds minimal expense compared to the potential costs of incomplete resection, positive margins requiring re-operation, or complications from inadequate exposure [11]. Furthermore, by potentially reducing operative time through improved exposure and eliminating the need for repeated retractor repositioning, this technique may offer indirect cost savings.

Several technical considerations merit attention. Appropriate sizing of the retractor is important; a small device (working diameter 2.5-6 cm) is typically suitable for perianal applications, selected based on incision length and estimated tumor depth. The initial dissection must create adequate space for retractor placement without compromising oncologic principles; the device should be introduced after limited dissection sufficient only to identify the sphincter anatomy and establish a safe tissue plane, so that its exposure and barrier function are available throughout the intersphincteric dissection. Surgeons should be mindful that excessive tension on the retractor rings could theoretically cause pressure necrosis of wound edges, though we have not observed this complication in our experience. A summary of practical recommendations and potential pitfalls is provided in Table 1.

**Table 1:** Tips and pitfalls for use of the Alexis® wound retractor in perianal oncologic surgery

Topic	Recommendation
Device sizing	Select small size (working diameter 2.5–6 cm) for most perianal applications. Match size to incision length and estimated tumor depth. An undersized device will not seat adequately; an oversized device will distort wound edges.
Timing of insertion	Insert after limited initial dissection sufficient to identify sphincter anatomy and establish a safe tissue plane, not before and not after complete circumferential dissection. Early insertion maximizes exposure and barrier benefit throughout the procedure.
Inner ring insertion	Introduce in a folded configuration. Ensure the ring is seated below the deep tumor margin without violating the capsule before releasing. Lubrication of the ring facilitates atraumatic insertion.
Tension adjustment	Roll the outer ring gradually and incrementally. Avoid excessive tension; over-rolling can cause pressure ischemia or necrosis of wound edges. Reassess tension periodically as dissection deepens.
Sterility	Combine with an intraluminal betadine-soaked rectal sponge for dual-barrier protection against fecal contamination. Replace the sponge if displaced during dissection.
Indications	Perianal tumors amenable to sphincter-preserving local excision; intersphincteric dissection requiring circumferential exposure; cases where contamination risk is a primary concern.
Relative contraindications	Very small or superficial tumors adequately managed with simpler retraction; tumors with extensive transmural sphincter involvement, where sphincter preservation is not oncologically feasible, regardless of exposure method.

Limitations of this technique include the requirement for an incision large enough to accommodate the retractor, which may not be suitable for all perianal lesions. Very small or superficial tumors might be adequately managed with simpler approaches. Additionally, tumors with extensive involvement of the sphincter complex may not be amenable to sphincter-preserving techniques regardless of the retraction method employed. Patient selection remains critical, and this technique is best suited for appropriately selected cases where sphincter preservation is oncologically feasible. Most importantly, this report describes a single case, and the contribution of the Alexis retractor to the outcome cannot be isolated from other technical factors, including the bimanual dissection technique, the effect of neoadjuvant therapy on tumor consistency and plane definition, and individual surgeon experience. No direct comparison with alternative retraction methods was performed. The conclusions drawn here are therefore confined to technical feasibility and should be interpreted as preliminary, hypothesis-generating observations rather than validated outcome data. Prospective evaluation and additional case series are needed to establish the reproducibility and broader clinical benefit of this approach.

We do not claim that this technique is entirely original, as wound retractors have been used in various surgical contexts, including colorectal resection and perineal surgery [4,7]. However, we believe the specific application of the Alexis device to the intersphincteric plane in perianal oncologic surgery, combined with an intraluminal betadine-soaked sponge as a dual sterility strategy, deserves emphasis and may benefit other surgeons facing similar technical challenges. The technique is straightforward to implement and requires no specialized training beyond familiarity with the device.

## Conclusions

The Alexis wound retractor represents a practical and reproducible technical adaptation for perianal oncologic surgery. In the case reported, the device provided superior circumferential exposure of the intersphincteric operative field compared to conventional retraction methods, improved operative ergonomics by eliminating the need for repeated retractor repositioning, and served as a physical barrier against fecal contamination when combined with an intraluminal betadine-soaked sponge. These observations support the technical feasibility of this approach in appropriately selected cases where sphincter-preserving resection is oncologically feasible. No SSI occurred, and sphincter continence was preserved at discharge, though these outcomes reflect a single case and cannot be attributed to the retractor in isolation. The technique is straightforward to implement and requires no specialized training beyond familiarity with the device. Prospective evaluation and additional case series are needed to validate reproducibility, define optimal patient selection criteria, and establish whether the technical advantages observed translate into measurable clinical benefit.
